# Trajectories of child emotional and behavioural difficulties before and during the COVID‐19 pandemic in a longitudinal UK cohort

**DOI:** 10.1002/jcv2.70068

**Published:** 2025-11-12

**Authors:** Nicky Wright, Daphne Kounali, Elise Paul, Alex S. F. Kwong, Daniel Major‐Smith, Ilaria Costantini, Deborah A. Lawlor, Kapil Sayal, Helen Bould, Nicholas J. Timpson, Kate Northstone, Melanie Lewcock, Kate Tilling, Rebecca M. Pearson

**Affiliations:** ^1^ School of Psychology Manchester Metropolitan University Manchester UK; ^2^ Population Health Sciences University of Bristol Medical School Bristol UK; ^3^ Bristol NIHR Biomedical Research Centre Bristol UK; ^4^ Research Department of Behavioural Science and Health Institute of Epidemiology & Health University College London London UK; ^5^ MRC Integrative Epidemiology Unit at the University of Bristol Bristol UK; ^6^ Division of Psychiatry University of Edinburgh Edinburgh UK; ^7^ ALSPAC Population Health Sciences Bristol Medical School University of Bristol Bristol UK; ^8^ Division of Psychiatry & Applied Psychology School of Medicine University of Nottingham Medical School Nottingham UK

**Keywords:** child, covid‐19 pandemic, emotional and behavioural problems, mental health, prospective, trajectories

## Abstract

**Background:**

There are substantial age‐related changes in emotional and behavioural problems over childhood. In order to establish the impact of the Covid‐19 pandemic on child emotional and behavioural problems, longitudinal designs which take into account age‐related trends are needed. This study examined trajectories of children's emotional and behavioural difficulties both before and during the pandemic in a prospective birth cohort.

**Methods:**

Data were from 708 children from the third generation of a birth cohort study; the Avon Longitudinal Study of Parents and Children in England. The study population comprised of 708 children (median age at COVID data collection was 2.75 years [interquartile range 0.8, 4.4]) whose parents provided pre‐pandemic surveys and a survey between 26 May and 5 July 2020. Multi‐level mixed effects models with random intercepts and slopes examined whether children's trajectories of emotional and behavioural difficulties during the pandemic differ from those expected pre‐pandemic.

**Results:**

Children's emotional and behavioural difficulties trajectories pre‐pandemic increased during infancy peaking around the age of 2 and then declined throughout the rest of childhood. Pre‐pandemic, decline in difficulties scores after age 2 was 0.6 points per month; but reduced by 35% during the pandemic. This slower decline in scores translated to older children having difficulty scores higher than would be expected, during the pandemic. By age 8.5, there is a 9.5‐point difference in pre‐ and during‐pandemic scores (95% CI: 4.5–14.5). This represents an average 48% increase from scores expected at this age (pre‐pandemic mean score = 20; 95% CI: [15–25]). Results remained similar although somewhat attenuated after adjusting for parental anxiety.

**Conclusion:**

The COVID‐19 pandemic may be associated with greater persistence of emotional and behavioural difficulties after the age of 2 years.

## Key Points

**What's Known?**

- The existing relatively small evidence base including pre‐pandemic prospective data to examine Covid‐19 pandemic impact on children is mixed. In order to establish whether the pandemic has increased emotional and behavioural difficulties in children, analyses which consider normative age‐related changes in problems are needed.

**What's New?**

- Examining trajectories before and during the pandemic in a UK cohort of 1–13‐year olds showed that the pandemic was associated with an increase in emotional and behavioural difficulties from age 2 onwards, representing a 0.8 SD difference by age 8.5 years.

**What's Relevant?**

- The Covid‐19 pandemic has increased the rates of emotional and behavioural problems in UK children. Continued longitudinal research is needed to establish whether this increase has persisted.

## INTRODUCTION

The question of whether the Covid‐19 pandemic and associated public health measures, including school and nursery closures, lockdown, and social distancing, impacted population mental health has received considerable research attention. Initial findings were largely based on cross‐sectional surveys, but in order to establish whether there has been a *change* associated with the pandemic, longitudinal designs with measurement pre‐pandemic on well‐characterised cohorts are needed. A number of reviews and meta‐analyses of longitudinal studies of both adults (Patel et al., 2022; Robinson et al., 2022) and children and adolescents (Kauhanen et al., 2023; Madigan et al., 2022; Miao et al., 2023; Newlove‐Delgado et al., 2023) have concluded that the pandemic was associated with a slight to small increase in mental health problems, but note substantial heterogeneity. However, when examining change in mental health difficulties in children this must be considered alongside the backdrop of firstly, the naturally occurring developmental progressions of symptoms over childhood (Bongers et al., 2004; Leve et al., 2005), and second, rising mental health problems over time in children and adolescents (Vizard et al., 2020).

Comparatively less studies have examined pandemic impact on mental health in child aged samples, and existing reviews and meta‐analyses of longitudinal studies have reached conflicting conclusions about the impact of the pandemic on children compared to adolescents. In Madigan et al.'s (2022) meta‐analyses of longitudinal cohort‐based studies of children and adolescents, the increases found in anxiety and depression were smaller (‘slight’ compared to ‘small’ effect) in children compared to adolescents, although only 6/53 studies included child aged samples. Kauhanen et al.'s (2023) review of longitudinal and repeated cross‐sectional studies, which focused on a broader range of mental health outcomes and included 5/23 studies of children, also concluded evidence for change in mental health symptoms in younger children was weaker. Another meta‐analysis of 20 longitudinal studies (Miao et al., 2023) found no effects of age of sample on change in anxiety and depression, suggesting children and adolescents experienced similar declines in anxiety and depression. However, this review only included 3 studies of children, with the focus on anxiety and depression measures likely limiting the number of studies which were included. Newlove‐Delgado et al.'s (2023) review and meta‐analysis of 51 longitudinal studies (including eight child aged samples) included a broader range of outcomes. Evidence was mixed, but studies focusing on emotional/internalising problems and behavioural/externalising problems indicated that there was a small increase that was larger in children, with some differences depending on child versus parent‐report.

The limited existing evidence from the UK is mixed but overall suggests that the pandemic may be associated with an increase in child mental health problems. In a cohort of 168 children (mean age = 8.7 years) Bignardi et al. (2020) found increases in child‐reported depression symptoms, but not parent‐reported emotional problems, compared to 18 months before the first UK lockdown. In another UK study of children rated at risk for emotional problems by teachers (mean age = 7.8 years), parent‐rated total difficulties and emotional problems increased from 17 months pre‐pandemic to post‐lockdown (July–September 2020). In a UK birth cohort, Wright et al. (2021) found an increase in depression and behavioural problems in 11–12 years olds assessed immediately pre‐pandemic and 3 months post‐pandemic onset. In a subsequent analysis of this sample, the increase in depression was removed in girls after accounting for age‐related change, but became stronger for boys, and both boys and girls showed increases in behavioural problems. In the NHS digital repeated cross‐sectional surveys, 16% of 5–16 year olds were identified as having a probable mental health disorder during the pandemic, compared to 11% 4 years before the pandemic (Vizard et al., 2020). In the UK Co‐Space study of 2988 children, aged 4–16 years, reported increases in hyperactivity/inattention and conduct problems from the beginning of the pandemic to July 2020 (Waite et al., 2021).

Longitudinal studies from outside the UK focusing on younger children have returned mixed findings, with one study in Spain finding increases in emotional and behavioural problems in preschool aged children (Alonso‐Martínez et al., 2021) and another study in Spain finding no change (Giménez‐Dasí et al., 2020). One US study found no change in uncooperative behaviour and worry in preschool‐aged children (Gassman‐Pines et al., 2022). No changes were found in emotional and behavioural problems in preschool‐aged children in a Brazilian birth cohort (Murray et al., 2023), whereas an increase in emotional problems was found in preschool aged children in a birth cohort in South India (Sharp et al., 2024).

Substantial evidence from longitudinal studies across childhood indicates that there is a normative developmental progression of emotional and behavioural difficulties, with behavioural difficulties peaking at toddler age and decreasing from age 2 onwards (Fanti & Henrich, 2010; Gilliom & Shaw, 2004; Hill et al., 2006). When establishing the impact of the pandemic, these age ‐related changes must be taken into account to determine whether change in symptoms is related to pandemic impact or explained by a natural decrease in symptoms over childhood. Only one existing study focusing on early childhood accounted for age‐related trends in their analysis, and reported a small impact of the pandemic on emotional problems in a sample of 528 preschool aged children in South India (Sharp et al., 2024).

Quantifying the impact of the pandemic on child emotional and behavioural problems is important because emotional and behavioural difficulties in childhood have an impact on educational and family functioning in the short/medium term and in the longer term are strongly associated with mental health problems in adulthood which are costly and difficult to treat (Copeland et al., 2015). We use data from a questionnaire designed specifically for the COVID‐19 pandemic in children of different ages, nested within a UK open population cohort study of predominantly white European ethnicity. The cohort we used enabled us to derive trajectories from infancy to late childhood (age 6 months to 13 years) during the pandemic and compare them to the same trajectories over the same ages in the same population. This provides a more robust method for estimating how young children have been affected by the pandemic, which is crucial for determining the extent of impact and developing appropriate public health response.

We aim to examine whether the Covid‐19 pandemic was associated with age‐related trajectories of young children's emotional and behavioural difficulties compared with expected trends, based on pre‐pandemic data. In addition, we aim to examine whether parental anxiety is associated with change in child emotional and behavioural symptoms, both to account for potential bias in reporting and because existing evidence suggests parental anxiety may moderate the impact of the pandemic on child mental health (Khoury et al., 2021).

## METHODS

### Study design and participants

The Avon Longitudinal Study of Parents and Children (ALSPAC) is an ongoing longitudinal population‐based study that recruited pregnant women residing in the south‐west of England with expected delivery dates between 1 April 1991 and 31 December 1992 (Boyd et al., 2013; Fraser et al., 2012). The cohort consists of the original 13,761 mothers and their partners (G0) and their 14,901 children (G1) (Northstone et al., 2019). In 2012, ALSPAC began recruiting and collecting data on the next generation, G2, the children of the G1 participants and grandchildren of the originally recruited G0 women (Lawlor et al., 2019). Details of the age distribution according to data availability are provided in Tables S1A and S1B. G2 participants can join the study at any time (from early pregnancy onwards), through an open cohort (Lawlor et al., 2019). Therefore, the G2 children have a range of birthdates and ages at any wave of data collection (including the pre‐pandemic and COVID data collection used here). More details of the participants who completed the COVID‐19 survey are found here (The Avon Longitudinal Study of Parents and…|Wellcome Open Research).

Data are collected from both parents (at least one of whom is a G1 participant) and their children. The study website contains details of all data available through a fully searchable data dictionary (http://www.bristol.ac.uk/alspac/researchers/our‐data/). Ethical approval for the study was obtained from the ALSPAC Law and Ethics Committee and the Local Research Ethics Committees and written informed consent was provided.

ALSPAC rapidly deployed two online‐only questionnaires in response to the COVID‐19 pandemic; one during the initial lockdown phase (9 April to 15 May 2020), and the second when lockdown restrictions started to ease (26 May to 5 July 2020). Data were collected using REDCap (Research Electronic Data CAPture tools) (Harris et al., 2009), a secure web application for building and managing online data collection exercises hosted at the University of Bristol. As part of the second COVID‐19 questionnaire, G1 parents were asked to complete an assessment about their G2 children (with parents with multiple children completely a questionnaire about each child). More details on the development of the questionnaire can be found elsewhere (Northstone et al., 2020).

This study includes 1407 observations from 708 children (belonging to 522 families) with up to seven measures (six pre‐pandemic and one during COVID‐19). The mean number of measurements was 2.21 (SD = 1.32) and to be included children had to have at least one measure before or during the pandemic. The majority (*N* = 434; 83%) of the responding parents for this study was the mother. Pre‐pandemic data were collected between May 2012 and December 2018 (Lawlor et al., 2019) and questions about the COVID‐19 pandemic data were collected in June 2020. A flow chart outlining the sample selection is presented in Figure S1 and further details of measures, including the number at each age, are provided in Figures S2–S5, and Tables S1A and S2B.

### Measures of child emotional and behavioural difficulties

Parents who participated in the COVID‐19 survey completed one of two assessments regarding their child's feelings and behaviour since the first UK lockdown began (23 March 2020), depending on the child's age. Parents of children younger than 36 months (henceforth ‘younger children’) completed the mood and distractibility subscales of the Carey Infant Temperament Questionnaire (ITQ; Carey & McDevitt, 1978) and parents of those aged 36 months or older (‘older children’) completed the Revised Rutter Parent Scale for Preschool Children (Elander & Rutter, 1996). Measures which were the same as, or as close as possible to measuring the same underlying constructs, as the two used during the pandemic were selected for use in this study The scores from different measures were re‐scaled the different scores to overcome differences in scale ranges and the simplest way to create a common scale that does not interfere with the individual scale psychometric properties. Table 1 shows each measure used at age pre‐pandemic and during pandemic. Further information on each measure, including psychometric data, can be found in Appendix S1.

**TABLE 1:** Emotional and behavioural problem measures used at each age pre‐pandemic and during Covid‐19.

Child age	Pre‐pandemic measure	During COVID‐19 measure
6 months	Carey Infant Temperament Questionnaire (ITQ)—*Mood* & *Distractibility* subscales	Carey Infant Temperament Questionnaire (ITQ)—*Mood* & *Distractibility* subscales
24 months	Carey Toddler Temperament Questionnaire (TTQ)—*Mood* & *Distractibility* subscales	Carey Infant Temperament Questionnaire (ITQ)—*Mood* & *Distractibility* subscales
36 months	Emotionality Activity Sociability (EAS) Temperament Survey—*Emotionality, Activity, Shyness, Sociability*	Revised Rutter Parent Scale for Preschool Children—*Behavioural difficulties score*
48 months	Revised Rutter Parent Scale for Preschool Children—*Behavioural difficulties score*	Revised Rutter Parent Scale for Preschool Children—*Behavioural difficulties score*
60 months	Emotionality Activity Sociability (EAS) Temperament Survey—*Emotionality, Activity, Shyness, Sociability*	Revised Rutter Parent Scale for Preschool Children—*Behavioural difficulties score*
72 months	Emotionality Activity Sociability (EAS) Temperament Survey—*Emotionality, Activity, Shyness, Sociability*	Revised Rutter Parent Scale for Preschool Children—*Behavioural difficulties score*
84 months	Strengths & Difficulties Questionnaire (SDQ)—*Total difficulties score*	Revised Rutter Parent Scale for Preschool Children—*Behavioural difficulties score*

#### Age 6 months (pre‐pandemic) and ages 0–36 months (COVID‐19 pandemic)

Summed scores of 19 parent‐completed items from the ‘Mood’ and ‘Distractibility’ subscales of the 99 item ITQ (Carey & McDevitt, 1978) were included.

#### Age 24 months (pre‐pandemic)

Parents were asked 19 questions about their child's recent behaviour from the ‘Mood’ and ‘Distractibility’ subscales of the Carey Toddler Temperament Questionnaire (TTQ; Fullard et al., 1984).

#### Age 48 months (pre‐pandemic) and ages 36 months and upwards (COVID‐19 pandemic)

Child emotional and behavioural difficulties were assessed using the behavioural difficulties score, which is a sum of the emotional difficulties, conduct difficulties, and the hyperactivity score from the Revised Rutter Parent Scale for Preschool Children (Elander & Rutter, 1996).

#### Ages 36, 60‐ and 72‐month pre‐pandemic

Parents completed the Emotionality Activity Sociability Temperament Survey for Children (Buss & Plomin, 1984) which is comprised of four subscales corresponding to traits described by Buss and Plomin. Items from these four scales were summed to form the total scale, with higher scores indicating more difficulties: emotionality, activity, shyness, and sociability.

#### Age 84 months pre‐pandemic

Twenty‐five items from the parent‐completed Strengths & Difficulties Questionnaire (SDQ; Goodman, 1997) were summed to form the total difficulties score. This score total score includes assessing different aspects of the child's mental health such as hyperactivity, conduct problems, and emotional difficulties.

### Measure of parental anxiety preceding the COVID‐19 pandemic

We adjusted for parental anxiety to reduce bias associated with parent reports of their children's emotional difficulties. We adjusted for anxiety rather than depression because our work on adult mental health in the parents found anxiety, rather than depression, increased during the pandemic (Kwong et al., 2020). The most recently assessed before COVID‐19 (age 24) measure of parental anxiety symptoms assessed using the Generalised Anxiety disorder symptoms using a computerised version of the Clinical Interview Schedule‐Revised (CIS‐R; Lewis et al., 1992) was included. We transformed this variable to an SD scale where 1 unit (1 SD) represents 3 points.

### Statistical analysis

We fitted two models using piecewise linear three‐level mixed effects with random intercept and slopes to estimate trajectories for the within‐child repeated measurements of emotional and behavioural difficulties over time (age), whilst accounting for clustering within families (Naumova et al., 2001). The modelling choice was primarily driven by substantive considerations. Pre‐pandemic observed trajectories (Figure S2) were consistent with previous findings where externalising behaviours are common in toddlerhood but become less common in the pre‐school years (Fanti & Henrich, 2010; Gilliom & Shaw, 2004; Hill et al., 2006). The equation for model 1 can be found in Appendix S2. In Model 1, our characterisation of child emotional and behavioural difficulties trajectories are summarised by four parameters: (i) the average score at age 2 where child difficulty scores peak, (ii) the rate of score change before, (iii) after 2 years, and (iv) a quadratic term to account for non‐linearity. To examine whether parental anxiety symptoms measured before the COVID‐19 pandemic explained any patterns in children's emotional and behavioural difficulties trajectories, we re‐estimated Model 1 but including pre‐pandemic parental anxiety (Model 2).

Within both models, we also examined the effect modification of ‘pandemic status’ on the trajectory parameters by including this as a two‐level grouping variable with two options referring to whether the measure was assessed before or during the pandemic. Variance according to child gender was considered in all models as a further grouping variable. Analyses were conducted using Stata 16 (StataCorp, 2019).

### Missing data

One‐hundred and thirty‐nine, 31% of the 522 parents, were missing parental anxiety measurements. We used multilevel multiple imputation using other measures of parental depression and anxiety that were collected pre‐pandemic. We used ‘jomo’ for multilevel joint modelling multiple imputation (Carpenter & Kenward, 2012). We pooled parameter estimates from three‐level mixed effect analyses adjusted for parental anxiety scores across 50 imputed datasets using Rubin's rules (Little & Rubin, 2019).

The objective of this study was to estimate the ‘total burden’ of the pandemic on children's emotional and behavioural well‐being. As COVID‐19 mitigation measure are a universal exposure, and thus does not vary according to socio‐demographic variables, we limited adjustments to parental anxiety and parental age (because it associated with missing data) only.

The children of all parent who participated in the two surveys were included. The majority of parents/responders (*N* = 503/708) had children with observations pre and during the pandemic, whilst there was also a group (*N* = 203/708) whose children were only observed during the pandemic, mostly because they were younger. We compared follow‐up time between these two groups and examined any dependence with observed key socio‐demographics including parental age, at child's birth, parental education, number of children, child's sex, parental anxiety. We also included the Index of Multiple Deprivation Quantile (IMD) which is a neighbourhood‐level indicator of relative poverty and is widely used in the UK. IMD classifies relative poverty in small geographical areas and not individuals and indicates the socioeconomic context individual people live in. IMD index compiles seven domains of deprivation, namely: income, employment, education, health, crime and barriers to housing and living environment (Abel et al., 2016).

The association between COVID‐19 survey response and key socio‐demographics was also explored using mixed effects Poisson regression.

## RESULTS

Demographic characteristics of study children and their parents are provided in Table 2. Most parents were mothers (83.1%), there was a relative even split of male and female children (47.03% female), and the median child age was 2.75 years (interquartile range 0.8–4.4 years). Most families had one child (70.31%). Parents mean age was 26.42 (SD = 1.84) and 19.5% educated to degree level or higher. Table S1A and S1B show the summary statistics for emotional and behavioural difficulties scores according to age Figure S2 depicts the observed trajectories of children's emotional and behavioural difficulties scores with age. Follow‐up patterns are also presented in Figure S2.

**TABLE 2:** Demographic characteristics of children and their parents during the COVID‐19 pandemic (*N* = 708).

Child characteristics	*N* (%)	Mean	SD	Min	Max
Child is female	333 (47.03)	‐	‐	0	1

		Median	IQR [25% 75%]		
Child age (years)—All observation	1407	2.7	[0.8 4.4]	0	13
Follow‐up time (years) (time elapse between first and last visit)	708	2.75	[0 4.9]	0	8.1
Age at first visit for those with both pre and post pandemic measurement (years)	503	0.5	[0.4 2.4]	0.25	7.5
Age of first visit for those with only post‐pandemic visit (years)	205	1.4	[0.7 2.2]	0	8.1

Parent characteristics	*N* (%)	Mean	SD	Min	Max
Parental sex (female)	434 (83.1)				
Number of children	522	1.3	0.59	1	6
1	367 (70.31)				
2	128 (24.52)				
≥3	27 (5.17)				
Parental age (at last visit), years		26.42	1.84	20.5	29.83
Parent education level^a^					
GCSE or lower	87 (16.67)				
Vocational training	132 (25.29)				
A‐level (high school diploma)	80 (15.33)				
University degree or higher	102 (19.54)				

Abbreviations: GCSE, General Certificate of Secondary Education; IQR, interquartile range.

^a^ Does not sum to 522 due to missing data.

**FIGURE 1:**
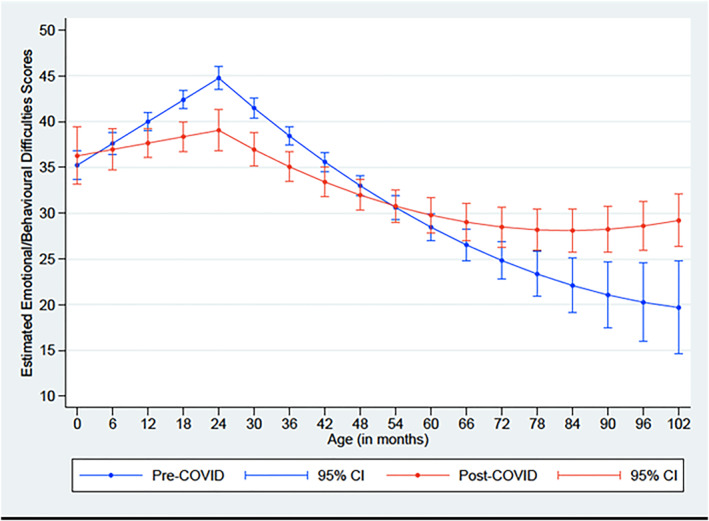
Estimates from the three‐level piecewise random effects (intercepts and slopes) model fitted to characterise emotional and behavioural difficulties score trajectories (*N* = 708 children).

**FIGURE 2:**
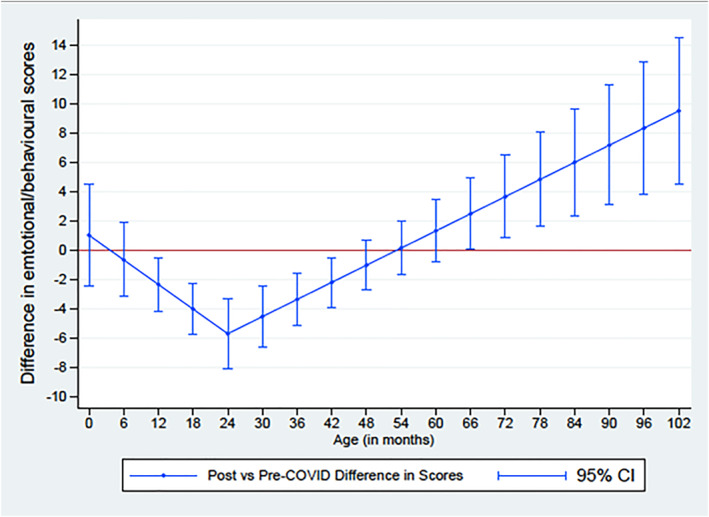
Unadjusted differences in post versus pre differences in children's emotional and behavioural difficulties scores.

Emotional and behavioural difficulties scores increase during infancy, peak around the age of 2 (24 months), and then decline during childhood at a decreasing rate of on average 0.565 (95% CI: 0.491–0.639) points per month (Table 3 and Figure 1). The peak difficulties scores at age 2 were lower during the COVID‐19 pandemic compared with pre‐pandemic scores for children at the same age. However, the rate of decline after age 2 was different according to pandemic status, where during‐pandemic trajectories drop by approximately one‐third as many points compared to pre‐pandemic trajectories (average rate of decline = 0.195 [95% CI: 0.113–0.277]) (Figure 2 and Table S3). By age 8.5, there are differences of approximately 10 points (95% CI: 5.00–15.00) between pre‐ and during‐pandemic scores, this represents a difference of 0.8 standard deviation.

**TABLE 3:** Model 1: estimates from the three‐level piecewise random effects (intercepts and slopes) model fitted to characterise emotional and behavioural difficulties score trajectories (*N* = 708 children).

Parameter	Fixed effect estimates			
	Mean	SD	*p*>|*z*|	[95% CI]
Emotional and behavioural difficulties characterisation pre‐pandemic				
Intercept: Score at 24‐month age^a^	44.758	0.638	<0.001	[43.508 to 46.008]
Pre‐24‐month rate—Linear (per month)^a^	0.396	0.045	<0.001	[0.308 to 0.485]
Post‐24‐month rate—Linear (per month)^a^	−0.565	0.038	<0.001	[−0.639 to −0.491]
Post‐24‐month rate—Quadratic^a^	0.003	0.001	<0.001	[0.002 to 0.004]
Pandemic effects				
Pandemic 24‐month score difference from pre‐pandemic score at 24 months	−5.704	1.212	<0.001	[−8.080 to −3.329]
Difference in infancy rate post‐pandemic from pre‐pandemic rate	−0.280	0.101	0.006	[−0.479 to −0.082]
Difference in childhood rate post‐pandemic from pre‐pandemic rate	0.195	0.042	<0.001	[0.113 to 0.277]

^a^ Reference comparison group: scores pre‐pandemic.

In Model 2, there was evidence for an association between parental anxiety and child trajectories (Table 3). For every 3‐point increase (or 1 SD increase) in parental anxiety there was on average 1.060 increase in children's difficulties scores (95% CI: 0.266–1.854). There was also evidence for a larger association between parental anxiety and trajectories during COVID‐19 than before COVID‐19 (Table S4). Adjustment for parental anxiety also attenuated differences in pre‐pandemic and during pandemic trajectories but there was still evidence for a difference according to pandemic status where during pandemic trajectories declined by 0.073 points fewer than pre pandemic trajectories (95% CI: 0.004–0.142) (Table 4 and Figure S3). We adjusted for parental age at child's birth in both model 1 (Table S5) and model 2 including parental anxiety (Table S6), which had little influence on the results. In the model accounting for possible child gender interactions, the pandemic effect remains unchanged (Table S7). There was some weak evidence that boys may have higher scores in infancy but no evidence of gender differences in the rates of change in infancy or childhood.

**TABLE 4:** Model 2: estimates from the three‐level piecewise random effects (intercepts and slopes) model fitted to characterise emotional and behavioural difficulties score trajectories adjusted for pre‐pandemic parental anxiety scores (*N* = 708 children).

Parameter	Fixed effect estimates			
	Mean	SD	*p*>|*z*|	[95% CI]
Emotional and behavioural difficulties characterisation pre‐pandemic				
Intercept: Score at 24‐month age^a^	44.412	0.644	<0.001	[43.150 to 45.673]
Pre‐24‐month rate—Linear (per month)^a^	0.375	0.046	<0.001	[0.284 to 0.466]
Post‐24‐month rate—Linear (per month)^a^	−0.604	0.037	<0.001	[−0.677 to −0.530]
Post‐24‐month rate—Quadratic^a^	0.005	0.001	<0.001	[0.004 to 0.006]
Pandemic effects				
Pandemic 24‐month score difference from pre‐pandemic score at 24 months	−3.476	1.166	0.003	[−5.762 to −1.191]
Difference in infancy rate post‐pandemic from pre‐pandemic rate	−0.139	0.095	0.143	[−0.324 to 0.047]
Difference in childhood rate post‐pandemic from pre‐pandemic rate	0.073	0.035	0.037	[0.004 to 0.142]
Parental anxiety scores				
Parental anxiety (SD scale: 1 SD = 3 units)	1.060	0.405	0.009	[0.266 to 1.854]

^a^ Reference comparison group: scores pre‐pandemic.

We found no evidence for associations of child's sex, and parental parity, multiple area deprivation index or parental education with completion of the COVID‐19 survey (Tables S8 and S9). Figures S4–S7 show missing data distributions by age and numbers of data points provided. Results were similar for a model with the sample with no missing data (Table S10).

## DISCUSSION

In this third generation of a unique longitudinal birth cohort study in the UK, we found that children's predicted trajectories of emotional and behavioural difficulties peaked around the age of 2 years, and then declined over the rest of early childhood. Emotional and behavioural difficulties in children under the age of two appeared to be lower during the pandemic than would be expected from pre‐pandemic trajectories. However, the expected decline after age two in emotional and behavioural difficulties was considerably attenuated in during‐pandemic trajectories compared to pre‐pandemic trajectories, resulting in more difficulties at older ages during than before the pandemic. The projected difference of 0.8 SD is large with clear clinical relevance given that children with psychological difficulties are substantially more likely to go on to have a mental health disorder in adulthood (Copeland et al., 2015).

The findings make an important contribution to the evidence base on the impact of the pandemic on emotional and behavioural difficulties in childhood. Existing reviews and meta‐analyses of longitudinal studies have reached inconsistent conclusions, but are based in small numbers of studies of children, particularly of younger children. By taking a more robust approach which examined age‐related trends in difficulties, the current study adds to a growing evidence base of studies which account for normative developmental change (Hosozawa et al., 2024; Sharp et al., 2024; Wright et al., 2024) and provides robust evidence that the pandemic was associated with an increase in emotional and behavioural difficulties in UK children. The UK experienced a mandated lockdown for nearly 2 months post‐onset of the pandemic, where all non‐essential businesses and schools were closed and restrictions were placed on leaving homes. Lockdown measures were then eased to allow people to return to work and subsequently return to school, but with 2 m social distancing rules and restrictions of gatherings in place. The Covid‐19 data collection in this study occurred during these periods of high restriction stringency. The lengthy and repeated school and nursery closures, which may result in a loss of structure and routine, support from school staff, time with peers, adequate nutrition, and physical activity may all have negatively impacted on child emotional and behavioural difficulties. Another publication from this sample suggested that loss of routines was associated with lower emotional and behavioural difficulties during the pandemic (Lees et al., 2023). This may be particularly relevant to why we observed an attenuation of the expected decline in emotional and behavioural difficulties, as more stability and consistency may be critical to emotion regulation capacities at this time.

We found that parental anxiety was associated with higher child trajectories of emotional and behavioural problems in general (i.e., irrespective of pandemic status), but accounting for parental anxiety led to some attenuation of results as expected. Consistent with these findings, a review of the association between the pandemic on mental health reported that parents of young children were disproportionately likely to affected by anxiety and depression symptoms (Aknin et al., 2022). This increased parental stress associated with the pandemic is in turn, also likely to negatively influence child mental health via parenting practices (Prime et al., 2020), as well as parental substance misuse and domestic violence (Holmes et al., 2020). A Spanish study conducted early in the pandemic found evidence that children's conduct and emotional problems were influenced by parents' perceived pandemic‐related distress mediated via parenting (Romero et al., 2020).

Limitations of the study include that our available sample size for comparing pre‐ to during‐pandemic child trajectories at very young ages was small and as a result, our estimates were less certain, as reflected in the wide confidence intervals. However, we had the benefit of up to seven repeated measures totalling more than >1000 observations which increased power. Nonetheless, a larger sample size would be needed to examine how different facets of children's functioning such as sub domains including hyperactivity or emotional problems, may be driving our findings and explore different items within the broader constructs.

A further limitation is that temperament scales were used at younger ages (0–3) and emotion difficulty scales at later ages (3+). Evidence shows these two constructs are strongly linked, however, there is a debate regarding the extent to which temperament is the same construct or a precursor of emotional difficulty (Egger & Angold, 2006; Lahey et al., 2004). Importantly, we have used equivalent constructs at each age in both pre and during the pandemic surveys. We also demonstrate that correlations between measures were similar to correlations between the same measure across ages. Thus, while caution is needed regarding interpretation of age‐related change (e.g., the decline from 2 years might in part be due to differences in the two scales) at both time points, differences between pre and during the pandemic scores should not be markedly influenced by differences in the scales. In addition, the scales used meant that we were not able to examine emotional and behavioural problems separately.

Given that the ALSPAC‐G2 children are offspring of an original cohort born in the same period of time from 1991 to 1992, the age of the child during the pandemic is intrinsically linked to the age of their mother or father at their birth. By definition, older children have mothers or fathers who were younger when they were born. For example, a mother or father of a child who turned 8‐years‐old in 2020 would have been 21–22 years old at their birth. In contrast, a mother or father of a child who turned 8‐years old in a 2017 pre‐pandemic assessment would have been 18–19 years old at their birth. Given that young parental age is a known risk factor for higher offspring emotional and behavioural problems (Chittleborough et al., 2011), this is likely to have meant that pre‐pandemic trajectories, especially for older children, would have been expected to be higher than during pandemic trajectories. This means if anything, the difference seen in older children during the pandemic in our study may be an underestimate. When we included parental age at birth of index child in the model this did not change the results. In addition, ALSPAC‐G2 recruitment is self‐selected. Recruitment into the cohort is ongoing, but the 2019 update reported that 57% of eligible pregnant G1 parents had consented into G2 by that point (Lawlor et al., 2019). ALSPAC G1 participants who consent into G2 do not differ from those who do not in terms of sex, educational attainment, smoking and whether they lived in the Bristol area at the time of recruitment, but do differ on BMI (within higher BMI in those consenting) and are more likely to have attended recent clinic assessments. The original ALSPAC sample is also primarily white European ethnicity and more affluent than other areas of the UK (Lawlor et al., 2019). This may limit generalisability to other, more diverse populations. Replication of our findings is important, though we are aware that few studies have the detailed repeat measures available in our study.

### Implications and conclusions

This was the first study of a UK child aged sample to take into account age‐related changes in emotional and behavioural difficulties when examining the impact of the Covid‐19 pandemic. The findings indicate the pandemic has reduced the expected age‐related decline in difficulties over childhood, representing a 0.8 SD difference by age 8.5 years. There are several implications for our findings. Firstly, our findings suggest that existing research with pre‐ and during pandemic comparisons, particularly where the pre‐pandemic measurement is not immediately prior to the onset of the pandemic, has likely underestimated the pandemic impact on children by not accounting for age‐related changes. This highlights how it is necessary to consider age‐related trajectories when examining the impact of discrete events over time, extending beyong investigations of Covid‐19 to all studies examining the impact of adverse events on mental health difficulties over childhood. Secondly, our findings demonstrate a potential greater persistence of emotional and behavioural difficulties from the age of two onwards during the pandemic with much larger than expected scores by older ages. Emotional and behavioural difficulties early in childhood are associated with psychiatric disorder in later childhood (Sayal et al., 2014) and late adolescence (Bould et al., 2014). If replicated, this could be an early indication of potential risk of increases in rates of childhood psychiatric disorder in the population. This increase is consistent with increases in referrals to Child and Adolescent Mental Health services observed between 2019 and 2023 (YoungMinds, 2023). Our findings have implications for policy and practice responses to address this increase in emotional and behavioural difficulties. Increased support and in particular, early intervention strategies, within schools, childcare, and community settings could help to reduce the effects of the pandemic. School‐ and community‐based interventions have shown some promise in reducing child mental health problems and allow support to be delivered on a larger scale (Fazel & Soneson, 2023). Continued longitudinal research on the impact of the COVID‐19 pandemic and its economic and social consequences will be necessary to determine if distress in children is maintained, as well as how presentations may change over time.

## AUTHOR CONTRIBUTIONS

**Nicky Wright**: Writing—original draft; writing—review and editing. **Daphne Kounali**: Investigation; writing—original draft; formal analysis; writing—review and editing; methodology; visualization; conceptualization. **Elise Paul**: Writing—review and editing; writing—original draft; investigation; methodology; formal analysis; conceptualization. **Alex S. F. Kwong**: Project administration; writing—original draft; investigation; writing—review and editing; conceptualization. **Daniel Major‐Smith**: Writing—review and editing; project administration. **Ilaria Costantini**: Writing—review and editing; methodology; formal analysis. **Deborah A. Lawlor**: Supervision; writing—review and editing; funding acquisition. **Kapil Sayal**: Project administration; writing—review and editing. **Helen Bould**: Writing—review and editing. **Nicholas J. Timpson**: Writing—review and editing; project administration; funding acquisition. **Kate Northstone**: Project administration; writing—review and editing; funding acquisition. **Melanie Lewcock**: Project administration; funding acquisition; data curation. **Kate Tilling**: Supervision; methodology. **Rebecca M. Pearson**: Conceptualization; writing—original draft; writing—review and editing; methodology; supervision; investigation.

## CONFLICT OF INTEREST STATEMENT

D. A. L. has received support from Medtronic Ltd and Roche Diagnostics for research unrelated to that presented here. The remaining authors have declared that they have no competing or potential conflicts of interest.

## ETHICAL CONSIDERATIONS

Written informed consent was obtained from participants and ethical approval for this study was obtained from the ALSPAC Ethics and Law Committee and the Local Research Ethics Committees under proposal B3503 in April 2020.

## Supporting information

Supporting Information S1

## Data Availability

The ALSPAC data for this project can be accessed through a system of managed open access. The steps below highlight how to apply for access to this ALSPAC data are: Please read the ALSPAC access policy which describes the process of accessing the data in detail, and outlines the costs associated with doing so. You may also find it useful to browse the fully searchable research proposals database, which lists all research projects that have been approved since April 2011. Please submit your research proposal for consideration by the ALSPAC Executive Committee. You will receive a response within 10 working days to advise you whether your proposal has been approved. If you have any questions about accessing data, please email alspac-data@bristol.ac.uk.
