# Supplementary material for: Trajectories of child emotional and behavioural difficulties before and during the COVID‐19 pandemic in a longitudinal UK cohort
Source: JCPP Adv. 2025 Nov 12;6(3):e70068. doi: 10.1002/jcv2.70068 (PMC13339118; doi:10.1002/jcv2.70068)
Supplement: Supplementary file 1 [file JCV2-6-e70068-s001.docx]

**Trajectories of child emotional and behavioural difficulties before and during the COVID-19 pandemic in a longitudinal UK cohort**

**Supporting Information**

**Appendix S1. Measures of child emotional and behavioural difficulties**

**Table S1A. Summary statistics for children’s pre-pandemic emotional and behavioural difficulties scores according to age**

**Table S1B. Summary statistics for children’s emotional and behavioural difficulties scores during the COVID-19 pandemic according to age**

**Table S2A. Distribution of observations according to pre-pandemic age when measurements were undertaken**

**Table S2B. Distribution of observations during the pandemic according to current age**

**Appendix S2. Model 1 Equation**

**Table S3. Unadjusted differences in post vs pre differences in difficulties scores**

**Table S4. Estimates from the three-level piecewise random effects**

**Table S5. Estimates from the three-level piecewise random effects (intercepts and slopes) model fitted to characterise emotional and behavioural difficulties score trajectories Adjusted for parental age at child’s birth (N = 708 children)**

**Table S6. Estimates from the three-level piecewise random effects (intercepts and slopes) model fitted to characterise emotional and behavioural difficulties score trajectories Adjusted for pre-pandemic parental anxiety scores and interaction with COVID-19 and parental age at child’s birth (N = 708 children contributing 1407 observations reported by 525 parents: after multiple imputation for parental anxiety)**

**Table S7. Model 1: Estimates from the three-level piecewise random effects (intercepts and slopes) model fitted to characterise emotional and behavioural difficulties score trajectories. (N = 708 children) adjusted for potential sex differences in trajectories**

**Table S8.** **Description of Key Socio-demographics characteristics according to response to the COVID survey**

**Table S9: Mixed effects Poisson Regression examining the dependence between missing rates and key child and parental sociodemographic characteristics including response to COVID survey**

**Table S10. Model 1: Estimates from the three-level piecewise random effects (intercepts and slopes) model fitted to characterise emotional and behavioural difficulties score trajectories for those children with were observed during the pandemic and also had pre-pandemic observations. (N= 188 children i.e., excluding those with no pre-covid or no-post-covid observation).**

**Figure S1. Sample flow chart of the total G2 sample of 746, which included all who had *either* pre or during COVID-19 data and excluded those with no pre or during COVID-19 data (n = 38).**

**Figure S2. Description of follow-up observations over time and observed trajectories of children’s emotional and behavioural difficulties.**

**Figure S3. Pandemic differences in children’s emotional and behavioural difficulties adjusted and unadjusted for parental anxiety.**

**Figure S4. Distribution of child age (years) during the pandemic. Greyed are the children who contribute observation during the pandemic n=393 and red is those who are in G2 but didn’t return the COVID survey n=315. This age also represents the follow-up time for those participating and contribute to our results. Participants in the COVID survey are also children with less follow-up time as there were not old enough to contribute but there is still considerable overlap. This is taken into account when examining rates of missing visits.**

**Figure S5. Distribution of child age (years) during the pandemic. Greyed are the children who contribute observation during the pandemic and pre-pandemic n=188, blue is those who are in G2 but didn’t return the COVID survey n=315 and red is those who returned the pandemic survey but do not have pre-pandemic data.**

**Figure S6. A description of the number of missing observations between those with and without observation pre-pandemic. The number of missing visits are calculated on the basis of visits a child could have had based on their age at the time of the G2 survey. For example, a child participating in the G2 survey who was 2 years old at the time of the survey could have had a maximum of two visits (could have participated in the pre-pandemic waves at 6 months) and no more than one missing observation.**

**Figure S7. A depiction of how missing rates are influenced by parental age between responders and non-responders to the COVID survey, where it can be seen how missing rates almost double with every year of parental age at birth of first child, along with the higher missing rates for non-responders. We found no evidence of a difference in the effect of parental age on missing rates between responders and non-responders. The IRR for missing rates according to a year increase in parental age is 2.15 (95% CI= [2.13 to 2.18]) among the children of survey responders vs 2.19 (95% CI= [2.17 to 2.22]) in the children of survey non-responders.**

**Appendix S1: Measures of child emotional and behavioural difficulties at scheduled visits according to age**

*Age 6 months (pre-pandemic) and ages 0-36 months (COVID-19 pandemic).* Summed scores of 19 parent-completed items from the ‘Mood’ and ‘Distractibility’ subscales from the Carey Infant Temperament Questionnaire (ITQ),^1^ 99 item were included. Parents rated items on a 5-point scale, adopted from the original 6-point scale. The summed score of all 19 items indicates more emotional and behavioural difficulties. Internal consistencies were acceptable at both ages (Cronbach’s α = 0.66 and 0.70).

*Age 24 months (pre-pandemic).* The Carey Toddler Temperament Questionnaire (TTQ)^2^ is designed for children aged 1-3 years and assesses nine temperamental characteristics. The 19 items included in the present study comprised the ‘Mood’ and ‘Distractibility’ subscales. The Mood (0.87) and Distractibility (0.69) subscales have been shown to have good test–re-test reliability.^2^ Parents rated statements on a 0-4 scale: almost never, rarely, sometimes, often, and almost always. As above, all 19 items were summed (Cronbach’s α= 0.64).

*Age 48 months (pre-pandemic) and ages 36 months and upwards (COVID-19 pandemic).* Twenty-seven items from the 42-item Revised Rutter Parent Scale for Preschool Children^3^ were included. The present study used the behavioural difficulties score, which is a sum of the emotional difficulties, conduct difficulties, and the hyperactivity score, plus 10 other items, for a total of 27 items. Possible scores range from 0-54 points, with higher scores reflecting more difficulties (Cronbach’s α = 0.86).

*Ages 36, 60- & 72-months pre-pandemic.* Parents completed the 42-item Emotionality Activity Sociability (EAS) Temperament Survey for Children^4^ at ages 60 and 72 months, and 20 items at age 36 months pre-pandemic. The EAS is comprised of four subscales corresponding to traits described by Buss and Plomin:^4^ emotionality (tendency to show distress), activity (preferred level of activity), shyness (tendency to be inhibited with unfamiliar people), and sociability (tendency to prefer the company of others). Items are rated between 0 (almost never) and 4 (almost always). The EAS is designed to be used with children aged between 1-9 years old. The EAS has showed good reliability and stability over time.^5^ Items were summed to form the total scale, with higher scores indicating more difficulties. Internal reliability for all three ages was good (Cronbach’s α = 0.74-0.89).

*Age 84 months pre-pandemic.* Parents completed the Strengths & Difficulties Questionnaire (SDQ)^6^ when children were 84 months old pre-pandemic. The SDQ consists of 25-items assessing different aspects of the child’s mental health such as hyperactivity, conduct problems, and emotional difficulties. Parents are given three response options, not true, somewhat true and true. Items were summed to form the total difficulties score (Cronbach’s α = 0.88).

We re-scaled child emotional and behavioural difficulties scores at different ages to a common scale (0-100) by dividing the sum score by the maximum possible in each scale and then multiplying by 100. See Tables S1A and S1B for basic descriptive information on each of the scales in original and “transformed” units.

Correlations between scales became stronger as age increased being 0.7-0.8 between ages 6, 7 and 8, and 0.3-0.4 between ages 2, 3, 4. The correlation between measures at age 3 (when a temperament scale was used) and age 4 (when an emotional/ behavioural scale was used) was larger than between ages 2 and 3 when temperament scales were used at both ages. This suggests the correlations across scales were similar to within scales at different ages.

**Table S1A. Summary statistics for children’s pre-pandemic emotional and behavioural difficulties scores according to age**

|  |  |  |  | **Pre-pandemic** | | | | | | | | |
| --- | --- | --- | --- | --- | --- | --- | --- | --- | --- | --- | --- | --- |
|  |  |  |  | **Emotional and Behavioural Difficulties Score** | | | | | | | | |
| **Measurement Wave** | | |  |  | **Original scale units** | | | **Transformed scale (0-100)** | | | |  |
|  | **Measure** | **Age interval**  **(months)**  **observed** | **Mean Age** | **SD**  **Age** | **No Items** | **Scale** | **Scale Max** | **N** | **Mean** | **SD** | **Min** | **Max** |
| 6 months | ITS | (0-18) | 5.49 | 0.99 | 19 | 4 | 76 | 290 | 37.71 | 11.09 | 6.79 | 64.47 |
| 24 months | TTQ | (18-28) | 23.73 | 1.56 | 19 | 4 | 76 | 205 | 47.12 | 9.87 | 12.47 | 73.68 |
| 36 months | EAS ^a^ | (28-35) | 31.63 | 2.18 | 20 | 4 | 80 | 40 | 36.93 | 8.97 | 15.00 | 55.26 |
| 48 months | RR | (35-53) | 41.33 | 5.86 | 27 | 2 | 54 | 273 | 33.50 | 12.53 | 1.85 | 65.00 |
| 60 months | EAS ^b^ | (53-60) | 57.77 | 1.86 | 42 | 4 | 168 | 48 | 27.16 | 8.98 | 11.90 | 46.30 |
| 72 months | EAS ^b^ | (60-70) | 61.65 | 2.50 | 42 | 4 | 168 | 52 | 30.48 | 11.47 | 7.55 | 55.36 |
| 84 months | SDQ | >70 | 75.55 | 5.53 | 25 | 3 | 75 | 106 | 28.11 | 12.40 | 6.67 | 68.00 |

Note. ITS = Carey Infant Temperament Scale.^1^ TTQ= Carey Toddler Temperament Questionnaire.^2^ EAS = Emotionality Activity Sociability Temperament Survey for Children.^4^ RR = Revised Rutter Parent Scale for Preschool Children.^3^ SDQ = Strengths and Difficulties Questionnaire.^6^ ^a^ Score contains 20 items from the EAS. ^b^ Score contains 42 items from the EAS.

**Table S1B. Summary statistics for children’s emotional and behavioural difficulties scores during the COVID-19 pandemic according to age**

|  |  |  | **During the COVID-19 pandemic** | | | | | | | | | | |
| --- | --- | --- | --- | --- | --- | --- | --- | --- | --- | --- | --- | --- | --- |
|  |  |  | **Emotional and Behavioural Difficulties Score** | | | | | | | | | | |
| **Measurement Wave** | | | | | **Original scale units** | | | **Transformed scale 0-100** | | | | | |
|  | **Measure** | **Age interval**  **(months)**  **observed** | **Mean**  **Age** | **SD**  **Age** | **No items** | **Scale** | **Scale**  **Max** | **N** | **Mean** | **SD** | **Min** | **Max** |  |
| 0-36 months | ITS | (0-18) | 8.93 | 5.31 | 19 | 4 | 76 | 112 | 37.25 | 9.95 | 12.60 | 57.89 |  |
| 0-36 months | ITS | (18-28) | 22.57 | 2.67 | 19 | 4 | 76 | 60 | 38.88 | 10.39 | 13.50 | 55.26 |  |
| 0-36 months | ITS | (28-35) | 31.00 | 2.02 | 19 | 4 | 76 | 23 | 34.53 | 12.43 | 14.47 | 56.09 |  |
| 36 + months | RR | (35-53) | 45.47 | 5.14 | 27 | 2 | 54 | 49 | 30.46 | 10.81 | 11.11 | 53.70 |  |
| 36 + months | RR | (53-60) | 56.73 | 1.86 | 27 | 2 | 54 | 22 | 30.14 | 13.02 | 9.26 | 55.56 |  |
| 36 + months | RR | (60-70) | 63.91 | 3.12 | 27 | 2 | 54 | 22 | 35.28 | 17.60 | 8.57 | 64.20 |  |
| 36 + months | RR | >70 | 99.17 | 21.91 | 27 | 2 | 54 | 105 | 32.40 | 15.18 | 5.56 | 75.93 |  |

Note. ITS = Carey Infant Temperament Scale ^1^. RR = Revised Rutter Parent Scale for Preschool Children ^3^

| **Pre-pandemic** | | | **Maximum number of observations** | | | | |
| --- | --- | --- | --- | --- | --- | --- | --- |
| **Age interval (months) observed** | **1** | **2** | **3** | **4** | **5** | **6** | **7** |
| (0-18) | 206 | 84 | 58 | 30 | 10 | 12 | <5 |
| (18-28) | 68 | 24 | 18 | 10 | 5 | <5 | <5 |
| (28-35) | 14 | <5 | <5 | <5 | <5 | <5 |  |
| (35-53) | 31 | 23 | 14 | 9 | <5 | <5 |  |
| (53-60) | 9 | <5 | <5 | <5 |  |  |  |
| (60-70) | 7 | 7 | <5 |  |  |  |  |
| >70 | 30 | 7 |  |  |  |  |  |

**Table S2A. Distribution of observations according to pre-pandemic age when measurements were undertaken
Table S2B. Distribution of observations during the pandemic according to current age**

| **During the pandemic** | | **Maximum number of observations** | | | | | |  |
| --- | --- | --- | --- | --- | --- | --- | --- | --- |
| **Age interval (months) observed** | **1** | **2** | **3** | **4** | **5** | **6** | **7** | |
| (0-18) | 206 |  |  |  |  |  |  | |
| (18-28) | 68 | 41 |  |  |  |  |  | |
| (28-35) | 14 | 17 | 5 |  |  |  |  | |
| (35-53) | 31 | 46 | 43 | 8 |  |  |  | |
| (53-60) | 9 | 7 | 7 | 10 |  |  |  | |
| (60-70) | 7 | 7 | 9 | 10 | <5 |  |  | |
| >70 | 30 | 33 | 31 | 27 | 17 | 19 | <5 | |

**Appendix S2: Model 1 Equation:**

The model assumes that score measurements depend upon the critical age of 24 months such that each subject has a baseline level and a pre- and post-critical age slope. If we denote the age at 24 months for each child j from responder/carer i, at each measurement occasion t, then

is the jth child score from parent i at each measurement occasion t

$=24-$ is the timing of the t^th^ measurement before and after the critical age.

is an indicator variable taking the of value 1 of the individual child measurement occasion is before the age of 24 months and 0 otherwise.

is an indicator variable taking the of value 1 of the individual child measurement occasion is taken before the lockdown and 0 otherwise.

is an indicator variable taking the of value 1 of the individual child is a girl and 0 if child is a boy.

$=+ + ++$	(1)

$+ +$	(2)

$+ +$	(3)

$+$	(4)

where,

$=+++$

$=++$

$=++$

is residual error (including measurement error normally distributed) and , k=0,1,2 for carer i and individual child j, are the random effects terms, independently and normally distributed and correspond to the intercept and pre- and post-critical age slopes, respectively and partitioning the different variance components corresponding to the level of carer/mother and individual child.

Part (1) of the model equation above correspond to over trajectory characterised by an intercept term representing the score level at the critical age of 24 months and pre- and post-critical age along with an additional quadratic term post critical age. These terms represent boys’ pre-pandemic.

Part (2) These terms represent how the intercept is modified by sex before and after the lockdown measures (interactions).

Part (3) These terms represent how the pre- and post-critical age slopes are modified by the pandemic in boys (interactions).

Part (4) These terms represent how the pre- and post-critical age slopes are modified by the pandemic in girls (interactions).

Adjustments for parental anxiety can be readily incorporated in the above equation as an additional predictor.

**Table S3. Unadjusted differences in post vs pre differences in difficulties scores taken from the model in Figure 2**

| **Age (years)** | **Mean Difference** | **SD** | **Low CI** | **High CI** |
| --- | --- | --- | --- | --- |
| 0 | 1.02 | 1.79 | -2.49 | 4.53 |
| 0.5 | -0.66 | 1.29 | -3.19 | 1.86 |
| 1 | -2.34 | 0.93 | -4.16 | -0.53 |
| 1.5 | -4.02 | 0.89 | -5.77 | -2.28 |
| 2 | -5.70 | 1.21 | -8.08 | -3.33 |
| 2.5 | -4.53 | 1.05 | -6.59 | -2.47 |
| 3 | -3.36 | 0.93 | -5.19 | -1.54 |
| 3.5 | -2.19 | 0.87 | -3.89 | -0.50 |
| 4 | -1.02 | 0.87 | -2.73 | 0.69 |
| 4.5 | 0.15 | 0.95 | -1.71 | 2.00 |
| 5 | 1.32 | 1.08 | -0.79 | 3.43 |
| 5.5 | 2.49 | 1.24 | 0.05 | 4.92 |
| 6 | 3.66 | 1.43 | 0.85 | 6.47 |
| 6.5 | 4.83 | 1.64 | 1.61 | 8.05 |
| 7 | 6.00 | 1.86 | 2.36 | 9.64 |
| 7.5 | 7.17 | 2.08 | 3.09 | 11.26 |
| 8 | 8.34 | 2.31 | 3.80 | 12.88 |
| 8.5 | 9.51 | 2.55 | 4.52 | 14.51 |

|  | **Fixed effect estimates** | | | | |
| --- | --- | --- | --- | --- | --- |
| **Parameter** | **Mean** | **SD** | **P>\|z\|** | **[95% CI]** | |
| **Emotional and behavioural difficulties characterisation pre-pandemic** | | | | | |
| Intercept: Score at 24-month age ^Ʇ^ | 44.446 | 0.644 | <0.001 | [43.184 | 45.708] |
| Pre-24-month rate – linear (per month) ^Ʇ^ | 0.375 | 0.046 | <0.001 | [0.284 | 0.466] |
| Post-24-month rate – linear (per month) ^Ʇ^ | -0.603 | 0.037 | <0.001 | [-0.676 | -0.530] |
| Post-24-month rate – quadratic ^Ʇ^ | 0.005 | 0.001 | <0.001 | [0.004 | 0.006] |
| **Pandemic effects** | | | | | |
| Pandemic 24-month score difference  from pre-pandemic score at 24 months | -3.514 | 1.169 | 0.003 | [-5.805 | -1.223] |
| Difference in infancy rate post-pandemic  from pre-pandemic rate | -0.145 | 0.095 | 0.126 | [-0.331 | 0.041] |
| Difference in childhood rate post-pandemic  from pre-pandemic rate | 0.072 | 0.035 | 0.040 | [0.003 | 0.141] |
| **Parental anxiety scores (SD scale 1 SD = 3 units)** |  |  |  |  |  |
| Parental anxiety effect pre-pandemic | 0.809 | 0.461 | 0.080 | [-0.096 | 1.714] |
| Parental anxiety effect post- pandemic | 1.716 | 0.695 | 0.014 | [0.352 | 3.081] |
| *Ʇ: Reference comparison group: Scores pre-pandemic* | | | | | |

**Table S4. Estimates from the three-level piecewise random effects (intercepts and slopes) model fitted to characterise emotional and behavioural difficulties score trajectories Adjusted for pre-pandemic parental anxiety scores and interaction with COVID-19 (N = 708 children).**

**Table S5. Estimates from the three-level piecewise random effects (intercepts and slopes) model fitted to characterise emotional and behavioural difficulties score trajectories Adjusted for parental age at child’s birth (N = 708 children)**

|  | **Fixed effect estimates** | | | | |
| --- | --- | --- | --- | --- | --- |
| **Parameter** | **Mean** | **SD** | **P>\|z\|** | **[95% CI]** | |
| **Emotional and behavioural difficulties characterisation Pre-pandemic** | | | | | |
| Intercept: Score at 24-month age ^Ʇ^ | 44.602 | 0.659 | <0.001 | [43.309 | 45.894] |
| Pre-24-month rate – linear (per month) ^Ʇ^ | 0.390 | 0.046 | <0.001 | [0.301 | 0.479] |
| Post-24-month rate – linear (per month) ^Ʇ^ | -0.575 | 0.039 | <0.001 | [-0.651 | -0.499] |
| Post-24-month rate – quadratic ^Ʇ^ | 0.003 | 0.001 | <0.001 | [0.002 | 0.005] |
| **Pandemic effects** | | | | | |
| Pandemic 24-month score difference  from pre-pandemic score at 24 months | -5.284 | 1.288 | <0.001 | [-7.809 | -2.759] |
| Difference in infancy rate post-pandemic  from pre-pandemic rate | -0.288 | 0.102 | 0.005 | [-0.487 | -0.089] |
| Difference in childhood rate post-pandemic  from pre-pandemic rate | 0.189 | 0.042 | 0.000 | [0.107 | 0.272] |
| **Parental age at child’s birth**  (SD scale 1 SD = 3 units) | -0.471 | 0.502 | 0.348 | [-1.455 | 0.513] |
| *Ʇ: Reference comparison group****:*** *Scores pre-pandemic* |  |  |  |  |  |

**Table S6. Estimates from the three-level piecewise random effects (intercepts and slopes) model fitted to characterise emotional and behavioural difficulties score trajectories Adjusted for pre-pandemic parental anxiety scores and interaction with COVID-19 and parental age at child’s birth (N = 708 children contributing 1407 observations reported by 525 parents: after multiple imputation for parental anxiety)**

|  | **Fixed effect estimates** | | | | |
| --- | --- | --- | --- | --- | --- |
| **Parameter** | **Mean** | **SD** | **P>\|z\|** | **[95% CI]** | |
| **Emotional and behavioural difficulties characterisation pre-pandemic** | | | | | |
| Intercept: Score at 24-month age ^Ʇ^ | 44.356 | 0.658 | <0.001 | [43.066 | 45.646] |
| Pre-24-month rate – linear (per month) ^Ʇ^ | 0.371 | 0.047 | <0.001 | [0.280 | 0.463] |
| Post-24-month rate – linear (per month) ^Ʇ^ | -0.609 | 0.038 | <0.001 | [-0.684 | -0.534] |
| Post-24-month rate – quadratic ^Ʇ^ | 0.005 | 0.001 | <0.001 | [0.004 | 0.006] |
| **Pandemic effects** | | | | | |
| Pandemic 24-month score difference  from pre-pandemic score at 24 months | -3.279 | 1.223 | 0.007 | [-5.676 | -0.882] |
| Difference in infancy rate post-pandemic  from pre-pandemic rate | -0.148 | 0.095 | 0.119 | [-0.334 | 0.038] |
| Difference in childhood rate post-pandemic  from pre-pandemic rate | 0.069 | 0.036 | 0.053 | [-0.001 | 0.139] |
| **Parental anxiety scores**  **(SD scale 1SD=3 units)** |  |  |  |  |  |
| Parental anxiety effect pre-pandemic | 0.767 | 0.468 | 0.102 | [-0.151 | 1.685] |
| Parental anxiety effect post-pandemic | 1.678 | 0.699 | 0.016 | [0.307 | 3.048] |
| **Parental Age at child’s birth**  (SD scale 1SD=3 units) | -0.316 | 0.483 | 0.513 | [-1.263 | 0.631] |
| *Ʇ: Reference comparison Group****:*** *Scores pre-pandemic* |  |  |  |  |  |

**Table S7. Model 1: Estimates from the three-level piecewise random effects (intercepts and slopes) model fitted to characterise emotional and behavioural difficulties score trajectories. (N = 708 children) adjusted for potential sex differences in trajectories**

|  | Fixed effect estimates | | | | |
| --- | --- | --- | --- | --- | --- |
| **Parameter** | **Mean** | **SD** | **P>\|z\|** | **[95% CI]** | |
| **Emotional and behavioural difficulties characterisation Pre-pandemic** | | | | | |
| Intercept: Score at 24-month age ^Ʇ^ | 45.621 | 0.782 | <0.001 | [44.089 | 47.153] |
| Pre-24-month rate – linear (per month) ^Ʇ^ | 0.446 | 0.057 | <0.001 | [0.334 | 0.559] |
| Post-24-month rate – linear (per month) ^Ʇ^ | -0.557 | 0.041 | <0.001 | [-0.638 | -0.476] |
| Post-24-month rate – quadratic ^Ʇ^ | 0.003 | 0.001 | <0.001 | [0.002 | 0.004] |
| **Pandemic effects** | | | | | |
| Pandemic 24-month score difference  from pre-pandemic score at 24 months | -5.652 | 1.209 | <0.001 | [-8.021 | -3.283] |
| Difference in infancy rate post-pandemic  from pre-pandemic rate | -0.280 | 0.101 | 0.006 | [-0.478 | -0.081] |
| Difference in childhood rate post-pandemic  from pre-pandemic rate | 0.190 | 0.042 | <0.001 | [0.109 | 0.272] |
|  |  |  |  |  |  |
| **Pre-pandemic gender differences** |  |  |  |  |  |
| Girl 24-month score difference  From boys at 24 months | -1.907 | 1.002 | 0.057 | [-3.872 | 0.057] |
| Girl infancy rate difference  From boys at 24 months | -0.109 | 0.078 | 0.163 | [-0.262 | 0.044] |
| Girl childhood rate difference  From boys at 24 months | -0.010 | 0.038 | 0.798 | [-0.084 | 0.064] |
| *Ʇ: Reference comparison Group****: Scores pre-pandemic*** | | | | | |

**Table S8.** **Description of key socio-demographics characteristics according to response to the COVID survey**

|  | | **Participated**  **(N=393 children, reported by 272 parents)** | | | | | | **Non-Responders to 1st COVID-19 survey**  **(N=315 children, reported by 250 parents)** | | | | | | **Total (N=708 children Report by 522 parents)** | | | | | |
| --- | --- | --- | --- | --- | --- | --- | --- | --- | --- | --- | --- | --- | --- | --- | --- | --- | --- | --- | --- |
|  |  | **Total** | | | | | | **Total** | | | | | | **Total** | | | | | |
| **Child** | | **N** | **M** | **SD** | **P25** | **P50** | **P75** | **N** | **M** | **SD** | **P25** | **P50** | **P75** | **N** | **M** | **SD** | **P25** | **P50** | **P75** |
| Child's age at COVID survey | | 393 | 3.8 | 3.07 | 1.33 | 2.88 | 5.83 | 315 | 5.80 | 2.34 | 4.0 | 5.58 | 7.58 | 708 | 4.69 | 2.94 | 2.21 | 4.42 | 6.92 |
| Parental age at child's birth | | 393 | 24.3 | 3.13 | 23.0 | 25.0 | 27.0 | 315 | 22.01 | 2.43 | 22.0 | 20.0 | 24.0 | 708 | 23.28 | 3.06 | 21.0 | 24.0 | 26.0 |
|  |  |  | **N** | **%** |  |  |  |  | **N** | **%** |  |  |  |  | **N** | **%** |  |  |  |
| Child's sex (Female) | | 393 | 186 | 47.33 |  |  |  | 315 | 147 | 46.67 |  |  |  | 708 | 333 | 47.03 |  |  |  |
| **Parent** |  |  |  |  |  |  |  |  |  |  |  |  |  |  |  |  |  |  |  |
|  |  | **N** | **M** | **SD** | **P25** | **P50** | **P75** | **N** | **M** | **SD** | **P25** | **P50** | **P75** | **N** | **M** | **SD** | **P25** | **P50** | **P75** |
| Parental age at first birth | | 272 | 23.6 | 3.33 | 21.0 | 24.0 | 26.0 | 250 | 21.49 | 2.56 | 19.0 | 21.0 | 24.0 | 522 | 22.61 | 3.17 | 20.0 | 23.0 | 25.0 |
| Parental Anxiety (GAD) | | 203 | 2.02 | 2.76 | 0.0 | 1.0 | 3.0 | 156 | 2.39 | 3.05 | 1.00 | 0.00 | 4.00 | 359 | 2.18 | 2.89 | 0.00 | 1.0 | 3.0 |
|  |  |  | **N** | **%** |  |  |  |  | **N** | **%** |  |  |  |  | **N** | **%** |  |  |  |
| Parental Parity | 1 | 272 | 166 | 0.61 |  |  |  | 250 | 110 | 0.44 |  |  |  | 522 | 276 | 0.53 |  |  |  |
|  | 2 | 272 | 83 | 0.31 |  |  |  | 250 | 110 | 0.44 |  |  |  | 522 | 193 | 0.37 |  |  |  |
|  | 3 | 272 | 22 | 0.08 |  |  |  | 250 | 26 | 0.10 |  |  |  | 522 | 48 | 0.09 |  |  |  |
|  | 4 | 272 | 0 | 0.00 |  |  |  | 250 | 3 | 0.01 |  |  |  | 522 | 3 | 0.01 |  |  |  |
|  | 5+ | 272 | 1 | 0.00 |  |  |  | 250 | 1 | 0.00 |  |  |  | 522 | 2 | 0.00 |  |  |  |

|  | | **Participated**  **(N=393 children, reported by 272 parents)** | | | | | | **Non-Responders to 1st COVID-19 survey**  **(N=315 children, reported by 250 parents)** | | | | | | **Total (N=708 children Report by 522 parents)** | | | | | |
| --- | --- | --- | --- | --- | --- | --- | --- | --- | --- | --- | --- | --- | --- | --- | --- | --- | --- | --- | --- |
|  | **Statistic** |  | **N** | **%** |  |  |  |  | **N** | **%** |  |  |  |  | **N** | **%** |  |  |  |
| Education | GCSE or lower | 233 | 44 | 0.19 |  |  |  | 168 | 43 | 0.26 |  |  |  | 401 | 87 | 0.22 |  |  |  |
|  | Vocational | 233 | 65 | 0.28 |  |  |  | 168 | 67 | 0.40 |  |  |  | 401 | 132 | 0.33 |  |  |  |
|  | AS/A level | 233 | 52 | 0.22 |  |  |  | 168 | 28 | 0.17 |  |  |  | 401 | 80 | 0.20 |  |  |  |
|  | Degree or higher | 233 | 72 | 0.31 |  |  |  | 168 | 30 | 0.18 |  |  |  | 401 | 102 | 0.25 |  |  |  |
|  | **Statistic** |  | **N** | **%** |  |  |  |  | **N** | **%** |  |  |  |  | **N** | **%** |  |  |  |
| Index of Multiple Deprivation Quintile | 1 | 258 | 88 | 0.34 |  |  |  | 231 | 53 | 0.23 |  |  |  | 489 | 141 | 0.29 |  |  |  |
|  | 2 | 258 | 53 | 0.21 |  |  |  | 231 | 59 | 0.26 |  |  |  | 489 | 112 | 0.23 |  |  |  |
|  | 3 | 258 | 52 | 0.20 |  |  |  | 231 | 45 | 0.19 |  |  |  | 489 | 97 | 0.20 |  |  |  |
|  | 4 | 258 | 42 | 0.16 |  |  |  | 231 | 42 | 0.18 |  |  |  | 489 | 84 | 0.17 |  |  |  |
|  | 5 | 258 | 23 | 0.09 |  |  |  | 231 | 32 | 0.14 |  |  |  | 489 | 55 | 0.11 |  |  |  |

**Table S8.** **Description of key socio-demographics characteristics according to response to the COVID survey (cont’d)**

**Table S9**: **Mixed effects Poisson Regression examining the dependence between missing rates and key child and parental sociodemographic characteristics including response to COVID survey.**

| **Characteristic** | **Units** | **Reference** | **IRR^¥^** | **SE** | **P-value** | **95% CI** | |
| --- | --- | --- | --- | --- | --- | --- | --- |
| **Child** |  |  |  |  |  |  | |
| Child Sex | 0-1 | Male | 0.88 | 0.08 | 0.171 | [0.73 | 1.06] |
| **Parent** |  |  |  |  |  |  |  |
| COVID-Survey Non-Responder | 0-1 | Responded | 1.54 | 0.15 | <0.001 | [1.28 | 1.87] |
| Parental age  at first birth | years |  | 2.09 | 0.04 | <0.001 | [2.02 | 2.17] |
| Parent's sex | 0-1 | Male | 0.90 | 0.12 | 0.420 | [0.70 | 1.16] |
| Parity | 2 | 1 child | 1.20 | 0.12 | 0.075 | [0.98 | 1.47] |
|  | +3 |  | 1.04 | 0.17 | 0.786 | [0.76 | 1.43] |
| Parental education | Vocational | GCSE or lower | 0.81 | 0.10 | 0.098 | [0.63 | 1.04] |
|  | AS/A level |  | 0.89 | 0.13 | 0.427 | [0.67 | 1.19] |
|  | Degree or higher |  | 0.99 | 0.14 | 0.935 | [0.74 | 1.31] |
| Index of Multiple Deprivation Quantile | 2 | First Quantile | 0.86 | 0.12 | 0.281 | [0.66 | 1.13] |
|  | 3 |  | 0.84 | 0.12 | 0.201 | [0.64 | 1.10] |
|  | 4 |  | 1.09 | 0.16 | 0.573 | [0.81 | 1.45] |
|  | 5 |  | 0.91 | 0.16 | 0.577 | [0.65 | 1.27] |
| Parental Anxiety | 0-12 |  | 1.02 | 0.02 | 0.251 | [0.99 | 1.05] |
| *^¥^IRR: incidence rate ratio* | | | | | | | |

Missing rates are higher among non-responders in the COVID survey where non-responders have 1.5 times the missing rate of responders. Apart from parental age we did not find any strong evidence of association with other parental key demographics.

**Table S10. Model 1: Estimates from the three-level piecewise random effects (intercepts and slopes) model fitted to characterise emotional and behavioural difficulties score trajectories for those children with were observed during the pandemic and also had pre-pandemic observations. (N= 188 children i.e., excluding those with no pre-covid or no-post-covid observation)**

|  | Fixed effect estimates | | | | |
| --- | --- | --- | --- | --- | --- |
| Parameter | Mean | SD | P>\|z\| | [95% CI] | |
| Emotional and behavioural difficulties characterisation pre-pandemic | | | | | |
| Intercept: Score at 24-month age ^Ʇ^ | 45.014 | 1.019 | <0.001 | [43.017 | 47.011] |
| Pre-24-month rate – linear (per month) ^Ʇ^ | 0.325 | 0.073 | <0.001 | [0.182 | 0.468] |
| Post-24-month rate – linear (per month) ^Ʇ^ | -0.489 | 0.054 | <0.001 | [-0.595 | -0.383] |
| Post-24-month rate – quadratic ^Ʇ^ | 0.002 | 0.001 | 0.009 | [0.001 | 0.004] |
| Pandemic effects | | | | | |
| Pandemic 24-month score difference  from pre-pandemic score at 24 months | -5.098 | 2.183 | 0.020 | [-9.378 | -0.819] |
| Difference in infancy rate post-pandemic  from pre-pandemic rate | -3.574 | 6.488 | 0.582 | [-16.291 | 9.143] |
| Difference in childhood rate post-pandemic  from pre-pandemic rate | 0.198 | 0.067 | 0.003 | [0.066 | 0.329] |
| Ʇ: Reference comparison group: Scores pre-pandemic | | | | | |

N=746

With Pre-Covid N=503

With Post-Covid N=188

No Post-Covid N=315

No Pre-Covid N=243

With Post-Covid N=205

No Post-Covid N=38

**Figure S1. Sample flow chart of the total G2 sample of 746, which included all who had *either* pre or during COVID-19 data and excluded those with no pre or during COVID-19 data (n = 38).**

**Figure S2. Description of follow-up observations over time and observed trajectories of children’s emotional and behavioural difficulties.**

We studied 708 children (375 boys and 333 girls) with an average number of 2 measurements over time (Figure 1, Tables SB1-SB2) 522 parents completed the questionnaires, of which 83.14 (n=434) were women 70.3% (n=367) of these parents contributed a single child; n=128 (24.5%) contributed 2 children; n=25 (4.8%) contributed 3 children and another 2 parents contributing 4, and 6 children respectively.

These children contributed a total of 1407 measurements over a maximum of seven occasions (Figure S1, Table SB1-SB2). In Figure 1 (right panel) we depict the observed trajectories of temperament scores with age.

**Figure S3. Pandemic differences in children’s emotional and behavioural difficulties adjusted and unadjusted for parental anxiety.**

**Figure S4. Distribution of child age (years) during the pandemic. Greyed are the children who contribute observation during the pandemic n=393 and red is those who are in G2 but didn’t return the COVID survey n=315. This age also represents the follow-up time for those participating and contribute to our results. Participants in the COVID survey are also children with less follow-up time as there were not old enough to contribute but there is still considerable overlap. This is taken into account when examining rates of missing visits.**

**Figure S5. Distribution of child age (years) during the pandemic. Greyed are the children who contribute observation during the pandemic and pre-pandemic n=188, blue is those who are in G2 but didn’t return the COVID survey n=315 and red is those who returned the pandemic survey but do not have pre-pandemic data.**

**Figure S6. A description of the number of missing observations between those with and without observation pre-pandemic.** **The number of missing visits are calculated on the basis of visits a child could have had based on their age at the time of the G2 survey. For example, a child participating in the G2 survey who was 2 years old at the time of the survey could have had a maximum of two visits (could have participated in the pre-pandemic waves at 6 months) and no more than one missing observation.**

**Figure S7. A depiction of how missing rates are influenced by parental age between responders and non-responders to the COVID survey, where it can be seen how missing rates almost double with every year of parental age at birth of first child, along with the higher missing rates for non-responders. We found no evidence of a difference in the effect of parental age on missing rates between responders and non-responders. The IRR for missing rates according to a year increase in parental age is 2.15 (95% CI= [2.13 to 2.18]) among the children of survey responders vs 2.19 (95% CI= [2.17 to 2.22]) in the children of survey non-responders.**

References

1.	Carey WB, McDevitt SC. Revision of the infant temperament questionnaire. *Pediatrics*. 1978;61(5):735-739.

2.	Fullard W, McDevitt SC, Carey WB. Assessing temperament in one-to three-year-old children. *J Pediatr Psychol*. 1984;9(2):205-217.

3.	Elander J, Rutter M. Use and development of the Rutter parents’ and teachers’ scales. *Int J Methods Psychiatr Res*. 1996;6(2):63-78.

4.	Buss A, Plomin R. *Temperament: Early Developing Personality Traits Lawrence Erlbaum Associates*. Lawrence Erlbaum; 1984.

5.	Bould H, Joinson C, Sterne J, Araya R. The Emotionality Activity Sociability Temperament Survey: Factor analysis and temporal stability in a longitudinal cohort. *Personal Individ Differ*. 2013;54(5):628-633. doi:10.1016/j.paid.2012.11.010

6.	Goodman R. The Strengths and Difficulties Questionnaire: a research note. *J Child Psychol Psychiatry*. 1997;38(5):581-586.
